# The association between experiential avoidance, depressive symptoms, and suicidal ideation in hospitalized older adults with functional impairments: the moderating role of resilience

**DOI:** 10.3389/fpsyt.2025.1739518

**Published:** 2026-01-09

**Authors:** Qi Tang, Meiyi Tao, Jiahui Zhang, Miliang Zou, Xiaofen Wang

**Affiliations:** 1 School of Nursing, Aging Health Research Center, Hunan Normal University, Health Science Center, Changsha, Hunan, China; 2 Kiang Wu Nursing College of Macau, Macao, Macao SAR, China; 3 Hunan Provincial People’s Hospital, Changsha, Hunan, China; 4 Department of Oncology, The Second Xiangya Hospital of Central South University, Changsha, Hunan, China

**Keywords:** hospitalized older adults with functional impairments, experiential avoidance, depressive symptoms, resilience, suicidal ideation

## Abstract

**Background:**

Experiential avoidance (EA) has been linked to intensified negative emotions among hospitalized older adults experiencing functional impairment due to physical limitations and the loss of autonomy, and this is linked to elevated suicidal ideation (SI) risk. This study examined the association between EA and SI, proposing depressive symptoms as a mediator and resilience as a moderator.

**Methods:**

717 hospitalized older adults with functional impairments were recruited from four hospitals. Participants completed validated questionnaires assessing EA, SI, depressive symptoms, and resilience. Mediation and moderation analyses were conducted using Hayes’ PROCESS macro (Model 4, 7 and 14) in SPSS.

**Results:**

The study revealed a significant positive association between EA and SI among hospitalized older adults with functional impairments (*β*=0.164, *P*<0.01), with depressive symptoms demonstrating a partial mediating effect in this association (*β*=0.356, *P*<0.01). Resilience played a dual moderating role in the associations: the strength of the association between EA and SI is weaker with higher resilience (*β*=-0.003, *P*<0.05) and concurrently mitigated the association between depressive symptoms and SI (*β*=-0.012, *P*<0.05).

**Conclusion:**

EA is associated with SI in hospitalized older adults with functional impairments, with depressive symptoms exerting a partial mediating role in this association. Resilience moderated the associations such that it weakened both the direct link between EA and SI, as well as the link between depressive symptoms and SI. Healthcare professionals should implement targeted suicide prevention strategies, including interventions to reduce EA, depressive symptoms, and enhance resilience, thereby mitigating SI in older adults.

## 1 Introduction

With the changes in population structure and the intensification of the aging process, disability has become a major public health issue worldwide, with the number of older adults with functional impairments steadily increasing. Older adults with functional impairments are those who experience physical limitations or disabilities due to aging, illness, or other conditions, requiring assistance from others or becoming entirely dependent on others for daily activities (1). According to WHO statistics, the global number of older adults with functional impairments is projected to increase from 101 million between 2010 and 2050 to 277 million (2). In China, over 40 million elderly individuals had disabilities by 2023, with projections indicating that this number could exceed 60 million by 2030 and potentially reach 96 million by 2050 in the absence of effective interventions (3).

Elderly inpatients often experience severe declines in physical function due to disease progression or surgical complications, transitioning from a state of normal functioning to one of disability. This dependence on others for daily living, mobility, healthcare, and social interactions leads to substantial changes in lifestyle and living conditions. Such drastic changes can trigger negative emotions, including anxiety, depression, and loneliness, resulting in considerable psychological distress and, in some cases, suicidal ideation (4). Suicidal ideation (SI) refers to self-reported thoughts or plans of suicide and is considered a potential immediate precursor to suicide attempts or completed suicide (5). Wahab et al. reported that hospitalized elderly patients in general hospitals exhibited a 5.9% prevalence of SI, influenced by factors such as underlying medical conditions, treatment, and economic burden (6). Older adults with functional impairments appear to have an even higher prevalence of SI (7). For hospitalized older adults with functional impairments, the presence of SI may accelerate disease progression, prolong recovery, and, in severe cases, lead to suicidal behavior, imposing significant burdens on patients, their families, and society (8). However, to date, little is known about the association between psychological factors and SI in this population, as well as the underlying mechanisms involved.

Mann and colleagues proposed the “stress-vulnerability” model of suicide, which posits that suicide results from an interactive process involving individual vulnerability factors, protective factors, and risk factors (9). Disability undoubtedly represents a significant negative stressor. Experiential avoidance refers to an individual’s efforts to suppress, deny, or distort thoughts in order to escape negative experiences following stressful events (10). This form of avoidance has been identified as a vulnerability factor that is strongly associated with suicidal ideation (11). In hospitalized older adults with functional impairments, disability often leads to limited physical activity and dependence on others for daily living, which can generate feelings of low self-esteem and shame. Experiential avoidance may provide short-term relief from these unpleasant emotions (12), but it can also contribute to various mental health problems, including severe sleep disturbances, anxiety, and depression (13), thereby increasing the likelihood of suicidal ideation.

Experiential avoidance has been shown to be positively correlated with depression (14), a relationship that is particularly pronounced among hospitalized older adults with functional impairments. However, avoidance does not eliminate the negative experiences associated with disability; rather, it temporarily suppresses or distorts these painful experiences while requiring considerable effort to manage, control, and resist them (10). Once their coping resources are depleted and alternative emotional regulation strategies are lacking, previously suppressed negative experiences resurface, leading to feelings of helplessness and further exacerbating depressive symptoms (15).

Numerous prior studies have consistently demonstrated that depression is a stronger predictor of SI (16–19). This predictive role has been confirmed among elderly individuals residing in nursing (20). Factors such as fear of illness, reliance on dependent care, poor adaptation to the hospital environment, and insufficient family support can exacerbate depressive symptoms, thereby increasing the risk of SI (16, 17, 21). These factors may also be directly associated with patients’ experiential avoidance and SI, suggesting a potential link among experiential avoidance, depressive symptoms, and SI. However, to date, only one study has indicated that depression mediates the relationship between experiential avoidance and SI, and this evidence comes from a sample of college students (22). Whether a similar association exists among hospitalized elderly individuals with disabilities remains to be investigated.

Resilience refers to an individual’s capacity and dynamic process to adapt effectively in the face of stress and adversity (23). Studies have shown that resilience is a significant negative predictor of SI (24), and Dursun et al. further demonstrated that enhancing resilience can reduce levels of experiential avoidance (25). Elderly individuals with higher levels of resilience are more likely to perceive stress and adversity positively, gradually overcoming depressive symptoms, which in turn alleviates SI (26). These findings suggest that resilience may exert a regulatory effect on experiential avoidance, depressive symptoms, and suicidal ideation. However, to date, no studies have directly examined this relationship in hospitalized older adults with functional impairments.

**Figure 1:**
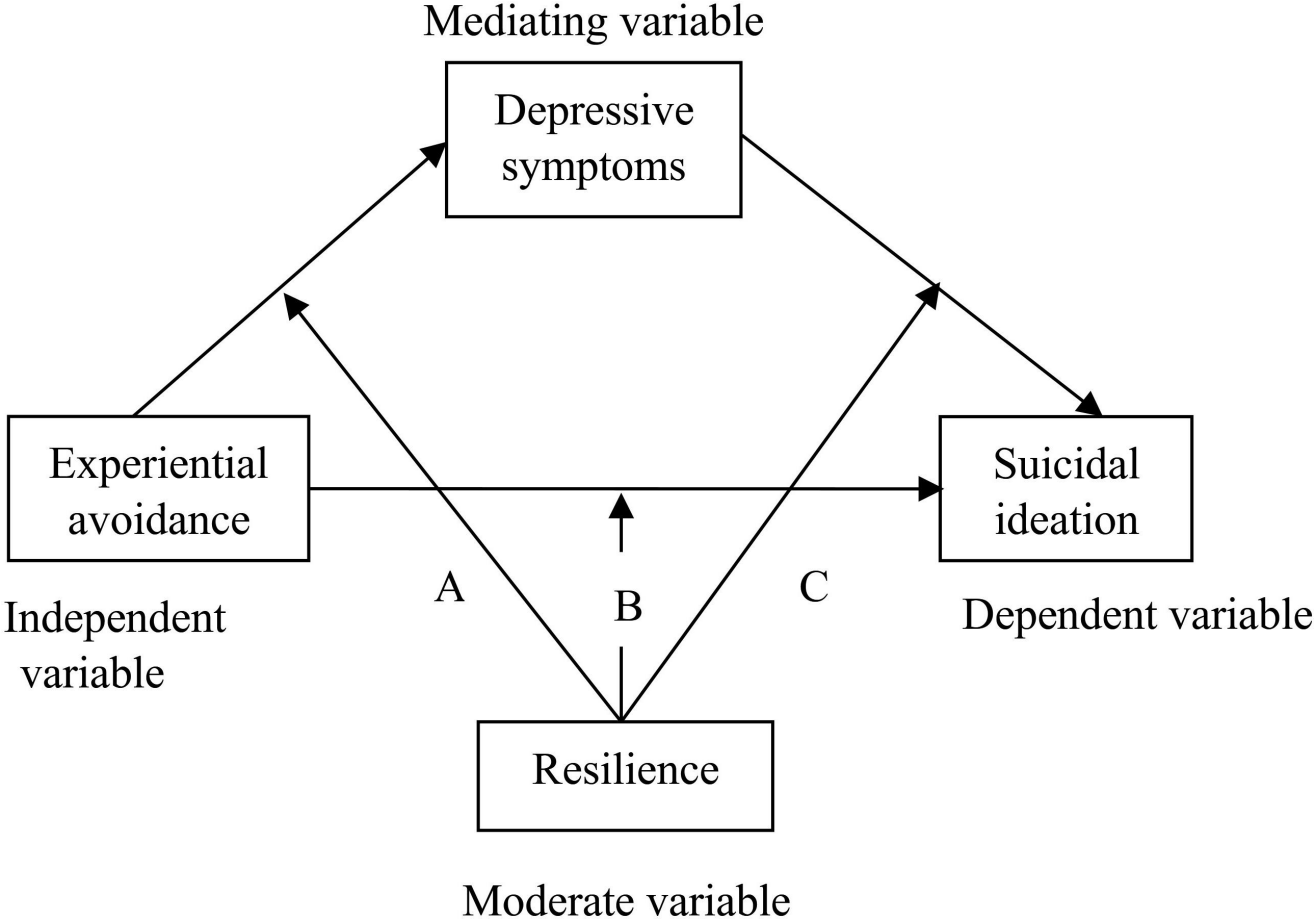
The conceptual framework of the hypothesis.

Based on the literature review, experiential avoidance can be considered a vulnerability factor for suicidal ideation, depression as a risk factor, and resilience as a protective factor. This study aims to construct a moderated mediation model among hospitalized older adults with functional impairments, exploring the mediating role of depression and the moderating role of resilience in the relationship between experiential avoidance and suicidal ideation (**Figure 1**).

## 2 Methods

### 2.1 Participants

This study recruited hospitalized older adults with functional impairments from four public tertiary hospitals in Hunan Province, China. A stratified sampling method was employed, wherein three clinical departments were first randomly selected from all departments containing elderly patients with functional impairments in each hospital, using a computer-generated random number sequence. Within these departments, all eligible patients admitted during the study period (June to October 2024) were invited to participate. The final sample constituted 717 participants. The sample size was calculated using the formula: $N=U_{a}^{2}P\left(1-P\right)/d^{2}$, The parameter *P* was derived from the reported 19.5% prevalence of SI among institutionalized elderly individuals in China (27), serving as a proxy measure given the absence of prior epidemiological data specific to hospitalized older adults with functional impairments (*P* = 0.195). with a consideration of a 20% invalidity/dropout rate, resulting in a minimum required sample size of 476 participants. The inclusion criteria were as follows: (1) age ≥ 60 years; (2) Barthel Index Rating Scale (BI) score < 100; (3) willingness to participate after informed consent. The exclusion criteria included: (1) patients with severe mental disorders accompanied by cognitive impairment; (2) functionally impaired patients who, following clinical evaluation by attending physicians, demonstrated rehabilitation potential for regaining activities of daily living (ADL) capacity through short-term therapeutic interventions (≤6 months duration). (3) patients unable to complete the survey. Trained graduate students distributed the questionnaires, ensured that each participant had signed an informed consent form, and guaranteed anonymity and independent completion of the questionnaires.

### 2.2 Instruments

#### 2.2.1 General demographic information

A self-developed demographic questionnaire was used to collect the participants’ age, gender, family residence, number of children raised, and relationship between caregivers and individuals, with a total of 15 items.

#### 2.2.2 Barthel index

The participants’ independence in daily activities was assessed via the Chinese version of the Barthel Index (BI). BI includes ten activities, such as eating, bathing, grooming, dressing, bowel and bladder control, toileting, bed and chair transfer, ambulation, and going up and down stairs (28). The cumulative score of all items varied between 0 and 100, with lower scores indicating worse ADL function. The total score was divided into 100 points for completely ADL independent, 76-99 points for mild disabled, 51-75 points for moderate disabled, 26-50 points for severely disabled, and 0-25 points for extremely severely disabled. The Cronbach’s α was 0.890 in the present study.

#### 2.2.3 Beck suicide ideation scale

SI was measured using the Suicide Ideation Scale originally developed by Beck et al. and has been validated with good reliability and validity in Chinese hospitalized older adults with the Cronbach’s α of 0.84 (4).BSS used 19 items to evaluate the subject’s suicidal ideation in the last week. When item 4 (i.e., active suicidal ideation) or item 5 (i.e., passive suicidal ideation) is answered with “weak” or “moderate to strong” (i.e., not 0), participants continue to finish the remaining items 6~19. Participants who scored ‘0’ on both items 4 and 5 were classified as not having SI and did not complete items 6-19, as per the scale’s skip pattern. Their total score was treated as 0 for the calculation of overall SI. The total scores are 0 to 38, with higher scores indicating stronger suicidal ideation. The Cronbach’s α for the BSS was 0.928 in this study.

#### 2.2.4 Acceptance and action questionnaire-II

Experiential avoidance was measured using the Acceptance and Action Questionnaire II (AAQ-II). The Chinese version of the AAQ-II was translated in 2013 and it has been validated with good reliability and validity in Chinese cancer patients with the Cronbach’s α of 0.94 (29). The AAQ-II consists of 7 items, rated on a 7-point Likert scale ranging from 1 (never true) to 7 (always true), with higher scores indicating greater experiential avoidance. The Cronbach’s α for the AAQ-II was 0.922 in this study.

#### 2.2.5 Geriatric depression scale – 15

The scale used in this study is a Chinese version and has been validated by Chinese scholars in hospitalized older adults (30). The Geriatric Depression Scale (GDS) was created with the express purpose of assessing depressive symptoms in older adults. The scale consists of a total of 15 items. In accordance with criteria, a score of ≥5 was used to indicate the presence of depressive symptoms. The higher the score, the higher the level of depression (31). The Cronbach’s α for the GDS-15 was 0.930 in this study.

#### 2.2.6 Connor-Davidson resilience scale

The Chinese version of the CD-RISC was used in the current study, which measures 25 items of five dimensions of resilience. The Cronbach’s α of this scale in the elderly population in Chinese hospitals is 0.93 (32). Participants were asked to respond to each item on a 5-point Likert scale, from 0 (not true at all) to 4 (true all the time). The higher the score, the higher the level of resilience. The Cronbach’s α for the CD-RISC was 0.980 in this study.

### 2.3 Ethic approval

The study protocol was approved by the Ethics Committee of Hunan Normal University (No. 2024-172). Written informed consent was obtained from all participants prior to enrollment, in accordance with the principles of the Declaration of Helsinki. Throughout the study, subjects’ emotions will be closely monitored. If significant fluctuations are observed, the study will be immediately suspended. Emotional support will be provided and the head nurse and chief physician notified. Resumption of the study will be determined based on emotional recovery. Persistent distress will necessitate referral to a mental health specialist for appropriate intervention.

### 2.4 Data analysis

Descriptive statistics and correlation analyses were conducted using SPSS 26.0. Mediation and moderated mediation models were tested using Hayes’ PROCESS macro (version 3.5), with gender and age as covariates. PROCESS Model 4 was used to test the mediation model, with experiential avoidance as the independent variable, SI as the dependent variable, and depression symptoms as the mediator. To investigate the potential moderating effect of resilience on the three pathways of ABC, PROCESS Model 59 was applied and further validated using PROCESS Model 7、14. Simple slope analyses were performed to clarify the moderating mechanism, and results were presented graphically. Continuous variables were centered, and standardized coefficients were reported. Bootstrap confidence intervals (95% CI) were calculated using 5,000 bootstrap samples, with statistical significance set at *P* < 0.05.

## 3 Results

### 3.1 Descriptive statistics

There were 717 participants, aged between 60 and 97 years (mean age=69.72+7.31 years; 56.2% males), completed the questionnaire and was included in the analysis. Among the participants, 17.4% reported suicidal ideation, 46.9% were from the city, 84.0% were married, 32.5% were disabled for the first time, and 17.3% were self-financed. For detailed information about the older adults, see **Appendix 1**.

### 3.2 Correlation analysis

The characteristics of the key variables and the results of the relevant analysis are shown in **Table 1**. EA was positively correlated with SI, while resilience was negatively correlated with both EA and SI. Depressive symptoms, which are considered a potential risk factor for SI, were positively correlated with it. In addition, depressive symptoms were also inversely associated with resilience.

**Table 1:** Correlation analysis.

Variables	1	2	3	4
1EA	–			
2Depression	0.738	–		
3SI	0.622	0.722	–	
4Resilience	-0.688	-0.799	-0.759	–

### 3.3 Testing the mediation effect

Based on correlation analysis, we performed a mediating effect analysis, and the results are shown in **Table 2**. EA was positively associated with depressive symptoms in Model 1(*β*=0.369, *P* < 0.01). In model 2, EA (*β*=0.164, *P* < 0.01) and depressive symptoms (*β* =0.965, *P* < 0.01) were both positively correlated with SI. The indirect effect of EA on SI through depressive symptoms was significant (*β*=0.356, Boot95%*CI*: 0.289-0.425), indicating that depressive symptoms partially mediated the relationship between EA and SI, accounting for 68.5% of the total effect.

**Table 2:** The mediation effect of depressive symptoms in the relationship between experiential avoidance and suicidal ideation (n=717).

	Depression (Model 1)					SI (Model 2)				
	*β*	*SE*	*t*	*LLCI*	*ULCI*	*β*	*SE*	*t*	*LLCI*	*ULCI*
Constant	-4.201	0.354	-11.886			-5.444	0.652	-8.355		
Age	0.023	0.018	1.278	-0.012	0.058	0.019	0.017	1.118	-0.014	0.052
Gender	-0.035	0.029	-1.207	-0.092	0.022	-0.028	0.025	-1.120	-0.077	0.021
EA	0.369	0.013	29.228^**^	0.344	0.393	0.164	0.031	5.218^**^	0.102	0.226
Depression						0.965	0.063	15.331^**^	0.841	1.088
R^2^	0.738					0.734				
F	854.294^**^					417.356^**^				

Bootstrap sample size = 5000. SE = Standard error. CI = Confidence interval.

Model 1: the relationship between EA and depression.

Model 2: the relationship between EA and SI with depression as mediator.

^*^P<0.05,^**^P<0.01.

### 3.4 Testing the moderated mediation effect

**Figure 2:**
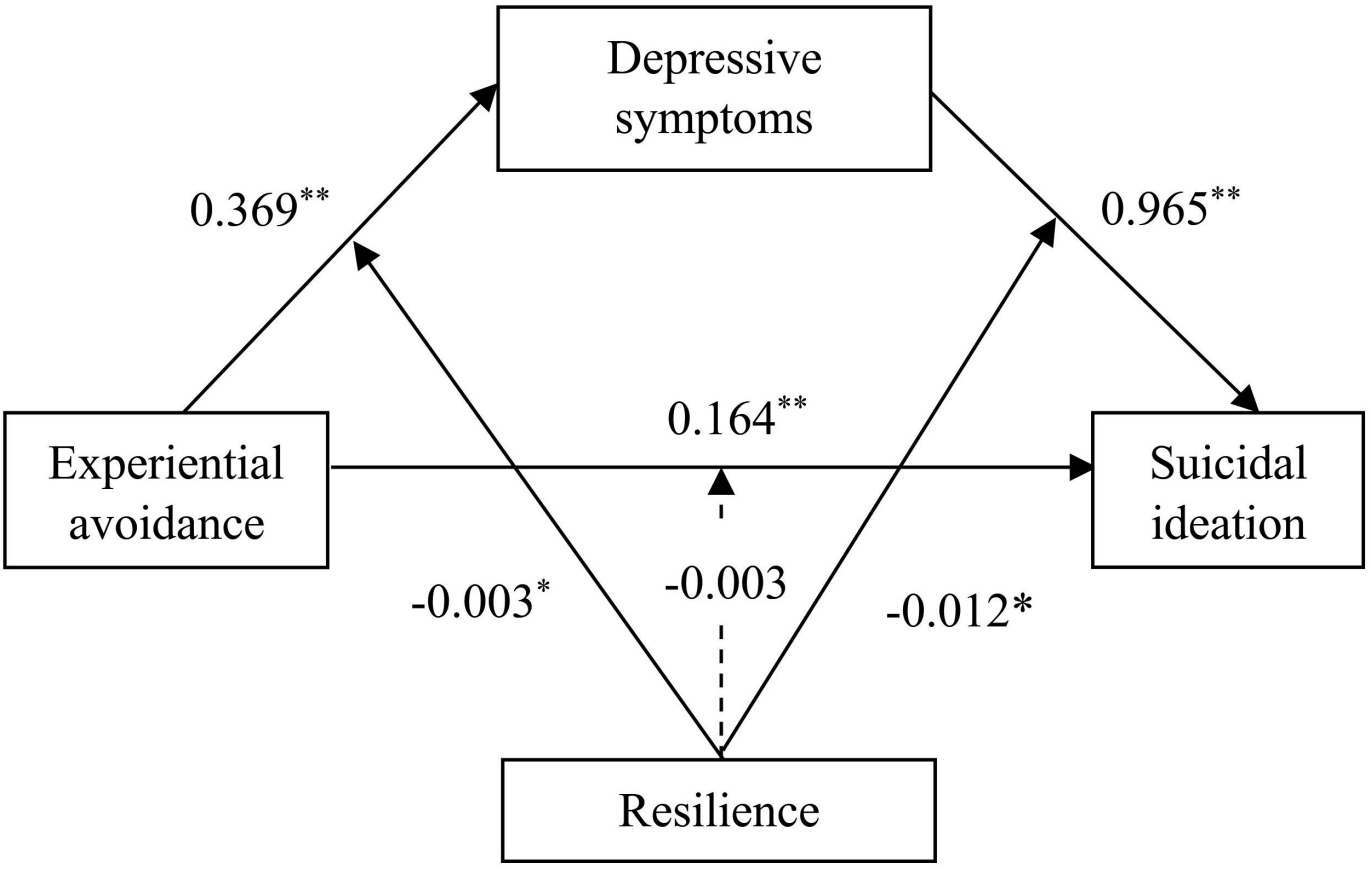
The moderated mediation model. *P<0.05, **P<0.01.

The analysis conducted using the SPSS PROCESS Model 59 revealed that, among the three pathways in which resilience was hypothesized as a moderating variable, only pathways A and B exhibited statistical significance (*P* < 0.05; **Figure 2**; **Table 3**). Further analysis using SPSS PROCESS Models 7 and 14 demonstrated that EA was positively correlated with depressive symptoms (*β*=0.423, *P* < 0.01), depressive symptoms were positively correlated with SI(*β* =1.142, *P* < 0.01), and the interaction between EA and resilience was negatively associated with depressive symptoms (*β*=-0.003, *P* < 0.05). Additionally, the interaction between depressive symptoms and resilience was negatively associated with SI (*β* =-0.012, *P* < 0.05). These findings confirm the moderating role of resilience in the relationships between EA and depressive symptoms, as well as between depressive symptoms and SI.

**Table 3:** The moderation effects (n＝717).

	Depression					SI				
	*β*	*SE*	*t*	*LLCI*	*ULCI*	*β*	*SE*	*t*	*LLCI*	*ULCI*
Constant	5.501	1.547	3.555			3.597	1.934	1.860		
EA(X)	0.423	0.044	9.656^**^	0.337	0.509	0.059	0.025	2.345^*^	0.010	0.108
Depression(M)						1.142	0.129	8.858^**^	0.889	1.395
Resilience(W)	-0.054	0.017	-3.152^*^	-0.088	-0.021	-0.046	0.019	-2.407^*^	-0.083	-0.008
X*W	-0.003	0.001	-5.727^*^	-0.004	-0.002					
M*W						-0.012	0.002	-8.074^*^	-0.015	-0.009
Age	0.021	0.016	1.312	-0.010	0.052	0.017	0.015	1.133	-0.012	0.046
Gender	-0.032	0.026	-1.231	-0.083	0.019	-0.025	0.023	-1.087	-0.070	0.020
R^2^	0.844					0.854				
F	586.868^**^					480.433^*^				

Bootstrap sample size = 5000. SE = Standard error. CI = Confidence interval.

Model 1: the moderating effect of resilience on the effect of EA on depression.

Model 2: the moderating effect of resilience on the effect of depression on SI.

^*^P<0.05,^**^P<0.01.

**Figure 3:**
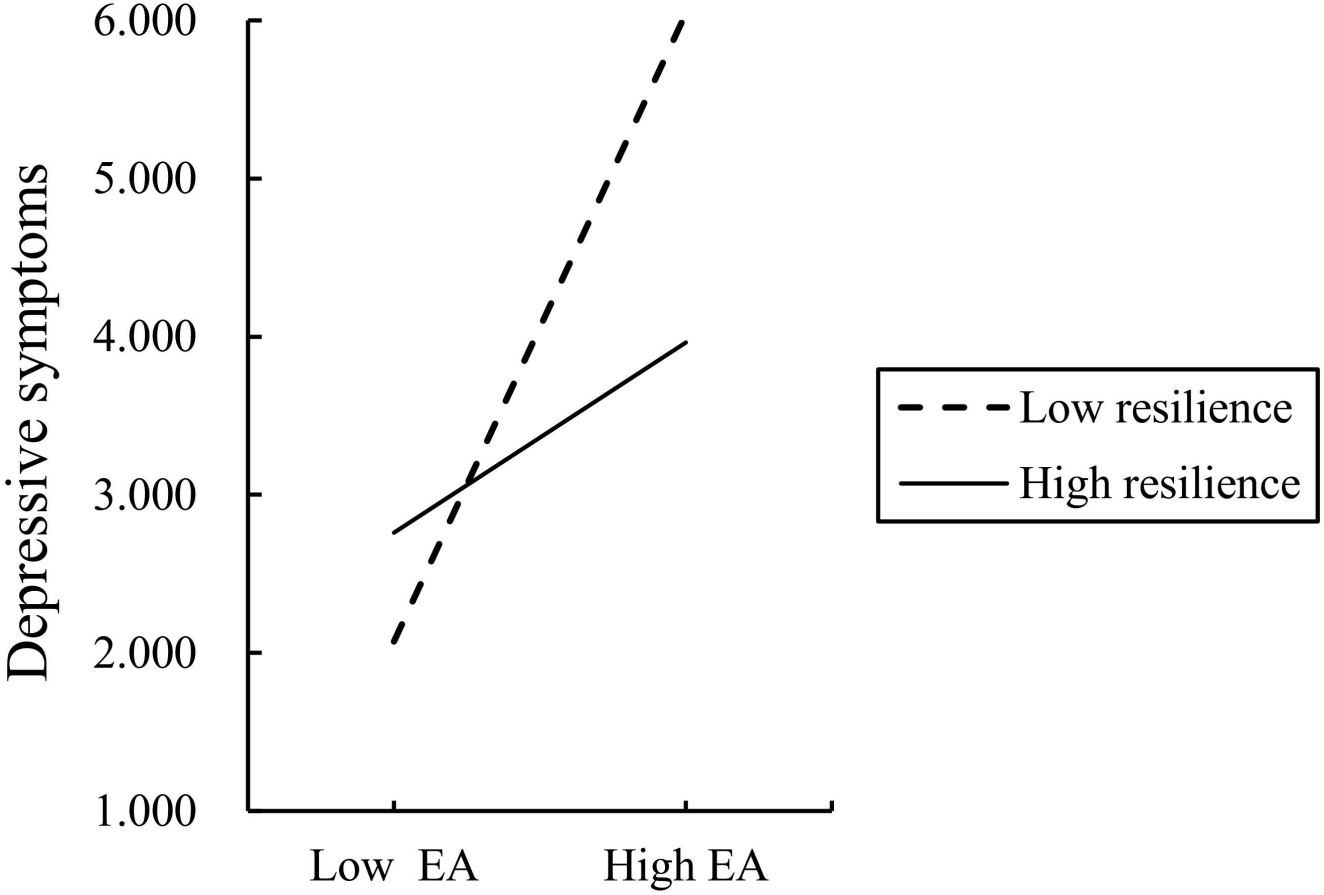
The moderating effect of resilience on the relationship between EA and depression.

**Figure 3** illustrates the association between EA and depressive symptoms at different levels of resilience. A significant positive association between EA and depressive symptoms was observed both when resilience was low (*β* = 0.205, *P* < 0.01) and high (*β* = 0.062, *P* < 0.001). However, the strength of this positive association decreased as the level of resilience increased.

**Figure 4:**
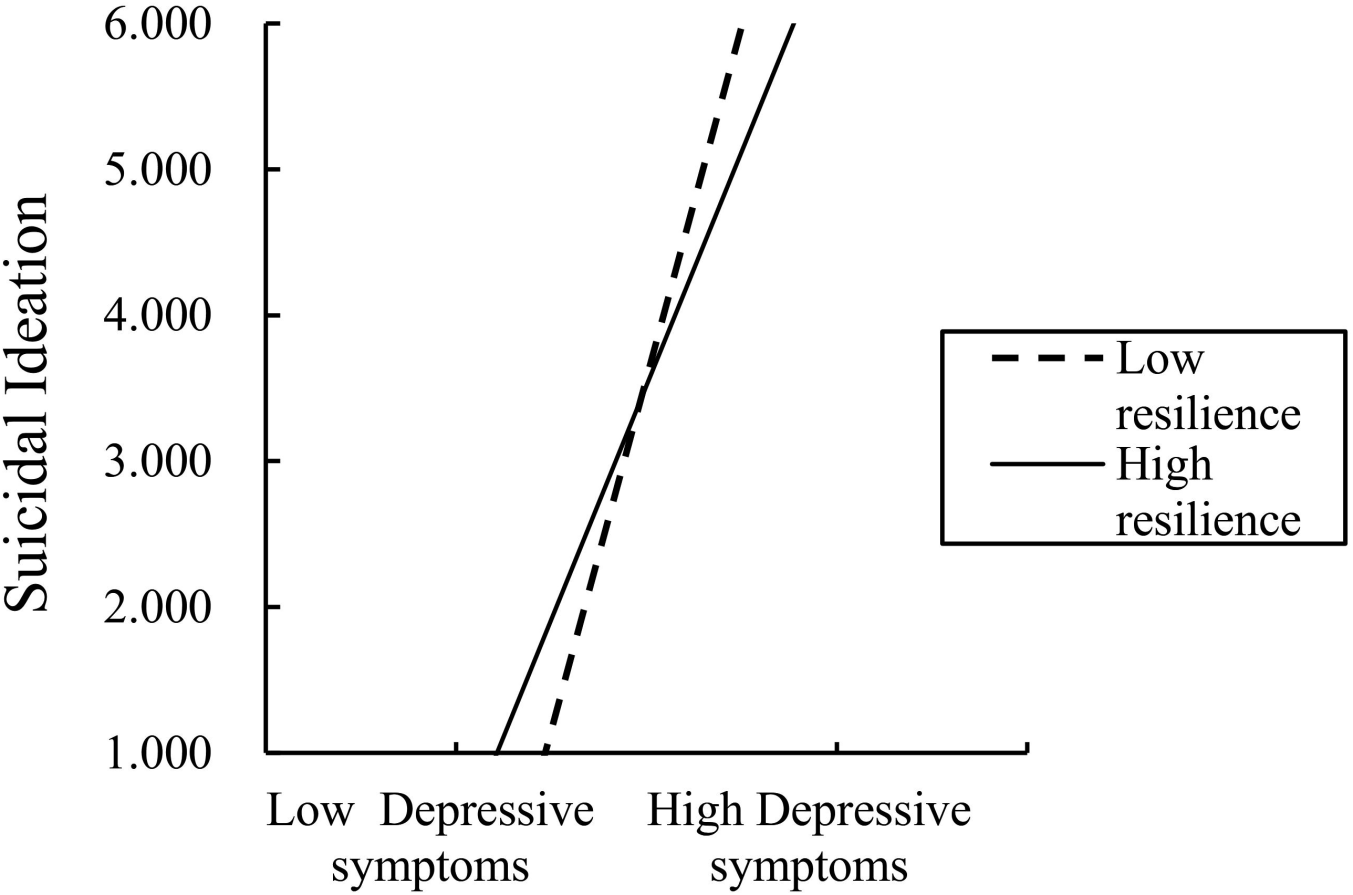
The moderating effect of resilience on the relationship between depression and SI.

**Figure 4** describes the association between depressive symptoms and SI at different levels of resilience. Depressive symptoms exhibited a significant positive association with SI both at low (*β* = 0.997, *P* < 0.01) and high (*β* = 0.662, *P* < 0.001) levels of resilience. Moreover, the strength of this association decreased as resilience increased.

## 4 Discussion

This study is the first to construct a moderated mediation model examining the relationship between experiential avoidance (EA) and SI among hospitalized older adults with functional impairments. The results indicate that EA is positively associated with both SI and higher levels of depressive symptoms in this population. Depressive symptoms serve as a potential mediating factor in the association between EA and SI. Furthermore, resilience weakens the associations between EA and depressive symptoms, as well as between EA and SI via the mediating role of depressive symptoms. Notably, as resilience increases, the levels of both depressive symptoms and SI decrease.

This study revealed a 17.4% prevalence of SI among hospitalized older adults with functional impairments, exceeding the rates reported in general hospitalized elderly populations (3.1%-8.8%) (4, 6), likely because those studies did not specifically focus on older adults with disabilities. While this prevalence is comparable to the 17.9% reported in Chinese nursing homes (33), it is lower than the 25.4% observed among nursing home residents with moderate to severe activity limitations (34). This difference may be attributed to the greater social isolation in nursing homes, whereas hospitalized patients benefit from more robust social support through healthcare interactions and family engagement during acute care (35). Accordingly, clinicians should implement structured psychosocial interventions, including proactive SI screening, therapeutic communication, and family education, to strengthen support networks and reduce risk.

Consistent with our hypothesis, higher levels of EA were positively associated with SI among hospitalized older adults with functional impairments. Faced with physical limitations, these individuals may engage in cognitive suppression or behavioral withdrawal to avoid disease-related distress (10). The restrictive hospital environment may further reinforce this tendency. While such avoidance may temporarily reduce discomfort, such avoidance is theorized to contribute to long-term emotional dysregulation, potentially establishing SI as a maladaptive coping mechanism (15). Acceptance and Commitment Therapy (ACT) has been shown to effectively reduce EA in clinical populations (36, 37). Accordingly, healthcare providers could guide patients to acknowledge negative thoughts without being controlled by them, fostering present-focused acceptance and adaptive emotional strategies to mitigate suicidal risk.

This study also identified a significant positive association between depressive symptoms and SI. This finding is consistent with prior research linking depressive symptoms to SI (16, 17, 19). Functional decline and dependency resulting from disability can undermine self-esteem, creating a vicious cycle that exacerbates psychological distress (18). Moreover, an uncertain prognosis may further diminish a sense of meaning, intensifying depressive symptoms. Mindfulness-based stress reduction (MBSR) has been shown to effectively alleviate depressive symptoms in patients with chronic conditions (38, 39). These findings suggest that healthcare providers could employ MBSR to help patients challenge negative self-perceptions (e.g., “disability equals worthlessness”), reduce depressive symptoms, and rebuild a sense of self-identity, thereby potentially lowering SI.

Additionally, the results suggested that depressive symptoms mediated the association between EA and SI, a finding consistent with previous research in other populations (40). Following the onset of disability, hospitalized older adults may face not only physical and economic challenges but also profound negative experiences like self-blame. The use of EA as a coping strategy, which involves sustained effort to suppress unwanted experiences, may intensify those same experiences and thereby contribute to the exacerbation of depressive symptoms (14). Higher levels of depressive symptoms are associated with increased attention to physical suffering and negative experiences, as well as more negative evaluations of stress and adversity. When these individuals feel overwhelmed, such patterns are linked to a heightened likelihood of SI (23).

Resilience moderated the association between EA and depressive symptoms and also indirectly influenced the association between EA and SI through its mediating role via depressive symptoms. This finding aligns with previous studies reporting that resilience moderates depressive symptoms and SI in other populations (41, 42). In this study, individuals with higher resilience appeared better able to draw on past experiences, mitigate hopelessness, and employ positive coping strategies, such as problem-solving and seeking social support, potentially reducing reliance on avoidance. This was associated with a lower accumulation of stress-related negative emotions and a reduced risk of depression (43). Furthermore, higher resilience was linked not only to a lower likelihood of depression but also to a weaker association between depressive symptoms and SI (34). Individuals with higher resilience may more effectively utilize external resources to manage negative experiences, which is associated with decreased SI (44). The American Psychological Association identifies five key strategies to build resilience: connection, wellness, purpose, healthy thinking, and seeking help (45). These strategies can be integrated into interventions, such as Mindfulness-Based Stress Reduction, by fostering patient communication, providing health education, enhancing self-worth through life reviews and goal-setting, and promoting gratitude and positive self-talk. Such multi-faceted approaches may strengthen resilience, improve emotion regulation, and are associated with reduced depression, anxiety, and suicidal risk.

Although the hypothesized moderated mediation model was supported by the data, it is important to consider alternative explanations for the observed associations, given the cross-sectional design of this study. For example, the relationships may be bidirectional: individuals with pre-existing suicidal ideation or depressive symptoms could be more likely to engage in experiential avoidance as a coping strategy to escape distressing thoughts and emotions. Consequently, future longitudinal or experimental studies are needed to clarify the temporal ordering and potential causal pathways among these variables.

### 4.1 Limitations

Firstly, due to the cross-sectional design of this study, causal relationships cannot be established. Secondly, given the sensitive nature of topics such as suicidal ideation addressed in this research, response bias may have occurred. Third, the selection of covariates was limited to age and gender, based on preliminary analyses indicating that other potential confounders were not significantly associated with the independent variable in this sample. Although this provides an empirical rationale, the exclusion of these factors remains a limitation. Finally, as the study was conducted solely in Hunan Province, the results may not be generalizable to other regions. Future studies could expand the sample to multiple locations, adopt longitudinal designs, and include a broader set of variables influencing suicidal ideation to better explore potential causal relationships.

## 5 Conclusion

Hospitalized older adults with functional impairments exhibited moderate levels of EA and SI, with a significant positive correlation between the two. Resilience was found to moderate the association between EA、depression symptoms and SI. These findings offer valuable insights for nursing staff and administrators seeking to reduce suicidal ideation and enhance resilience in this population. Future studies should further investigate the longitudinal relationships among experiential avoidance, depressive symptoms, and suicidal ideation to inform the development of evidence-based interventions.

## Data Availability

The raw data supporting the conclusions of this article will be made available by the authors, without undue reservation.
