# Supplementary material for: The association between experiential avoidance, depressive symptoms, and suicidal ideation in hospitalized older adults with functional impairments: the moderating role of resilience
Source: Front Psychiatry. 2026 Jan 9;16:1739518. doi: 10.3389/fpsyt.2025.1739518 (PMC12827635; doi:10.3389/fpsyt.2025.1739518)
Supplement: Supplementary file 1 [file SupplementaryFile1.docx]

Demographic information in the quantitative survey (N = 717)

| Characteristic |  | Total | Non-SI  N=（592） | SI  N=（125） | t/F | $P$ value |
| --- | --- | --- | --- | --- | --- | --- |
| Gender | Male | 336（46.9） | 295（49.8） | 41（32.8） | 10.458 | 0.001 |
|  | Female | 381（53.1） | 297（50.2） | 84（67.2） |  |  |
| Age | 61-70 | 420（58.6） | 349（59.0） | 71（56.8） | 32.579 | ＜0.001 |
|  | 71-80 | 229（31.9） | 187（31.6） | 42（33.6） |  |  |
|  | ＞80 | 68（9.5） | 56（9.4） | 12（9.6） |  |  |
| Ethnic group | Han nationality | 605（84.4） | 486（82.1） | 119（95.2） | 10.944 | 0.356 |
|  | Minority | 112（15.6） | 106（17.9） | 6（4.8） |  |  |
| Income | ≤3000 CNY | 119（16.6） | 5（0.9） | 114（77.6） | 103.563 | ＜0.001 |
|  | 3001~6000 CNY | 286（39.9） | 253（28.2） | 33（22.4） |  |  |
|  | 6001~9000 CNY | 225（31.4） | 225（31.4） | 0 |  |  |
|  | ＞9000 CNY | 87（12.1） | 87（39.5） | 0 |  |  |
| Education level | Primary and below | 268（37.3） | 200（33.7） | 68（54.4） | 9.434 | ＜0.001 |
|  | Middle school | 256（35.7） | 213（36.0） | 43（34.4） |  |  |
|  | High school | 80（11.2） | 72（12.2） | 8（6.4） |  |  |
|  | College and above | 113（15.8） | 107（18.1） | 6（4.8） |  |  |
| Marital status | Unmarried | 2（0.3） | 1（0.2） | 1（0.8） | 31.351 | 0.087 |
|  | Married | 602（84.0） | 530（89.5） | 72（57.6） |  |  |
|  | Widowed/divorced | 113（15.7） | 61（10.3） | 52（41.6） |  |  |
| Family relationships | Good | 376（52.4） | 352（59.5） | 24（19.2） | 160.562 | ＜0.001 |
|  | Ordinary | 294（41.0） | 231（39.0） | 63（50.4） |  |  |
|  | Bad | 47（6.6） | 9（1.5） | 38（30.4） |  |  |
| Pre-disabling working condition | Farmers | 377（52.6） | 292（49.3） | 85（68.0） | 8.104 | ＜0.001 |
|  | Incumbency | 11（1.5） | 7（1.2） | 4（3.2） |  |  |
|  | Retire | 329（45.9） | 293（49.5） | 36（28.8） |  |  |
| Type of disease | Cancer | 121（16.9） | 96（16.2） | 25（20.0） | 0.535 | 0.710 |
|  | Heart and brain | 394（55.0） | 330（55.7） | 64（51.2） |  |  |
|  | Blood disease | 111（15.5） | 90（15.3） | 21（16.8） |  |  |
|  | Accidental dismemberment | 56（7.8） | 48（8.1） | 8（6.4） |  |  |
|  | Other | 35（4.8） | 28（4.7） | 7（5.6） |  |  |
| Whether it is a first-time disability | Yes | 233（32.5） | 143（24.2） | 90（72.0） | 146.999 | ＜0.001 |
|  | No | 484（67.5） | 449（75.8） | 35（28.0） |  |  |
| Degree of disability | Extremely severely disabled | 60（8.4） | 41（6.9） | 19（15.2） | 88.283 | ＜0.001 |
|  | Severely disabled | 69（9.6） | 30（5.1） | 39（31.2） |  |  |
|  | Moderate disability | 119（16.6） | 71（12.0） | 48（38.4） |  |  |
|  | Mild disability | 469（65.4） | 450（76.0） | 19（15.2） |  |  |
| Payment Methods | Privately pay | 124（17.3） | 57（9.7） | 67（53.6） | 99.749 | ＜0.001 |
|  | Medical insurance for urban and rural residents | 314（43.8） | 288（48.6） | 26（20.8） |  |  |
|  | Employee medical insurance | 279（38.9） | 247（41.7） | 32（25.6） |  |  |
| Experiential avoidance | $M$±$SD$ | 26.34±9.69 | 23.68±8.08 | 38.93±6.13 | 16.758 | ＜0.001 |
| Depression | $M$±$SD$ | 5.50±4.84 | 3.96±3.52 | 12.80±3.34 | 68.826 | ＜0.001 |
| Resilience | $M$±$SD$ | 77.12±28.74 | 88.40±15.14 | 23.70±13.81 | 20.581 | ＜0.001 |
